# Scaffold and Structural Diversity of the Secondary Metabolite Space of Medicinal Fungi

**DOI:** 10.1021/acsomega.2c06428

**Published:** 2023-01-10

**Authors:** R.P. Vivek-Ananth, Ajaya Kumar Sahoo, Shanmuga Priya Baskaran, Areejit Samal

**Affiliations:** † The Institute of Mathematical Sciences (IMSc), Chennai600113, India; ‡ Homi Bhabha National Institute (HBNI), Mumbai400094, India

## Abstract

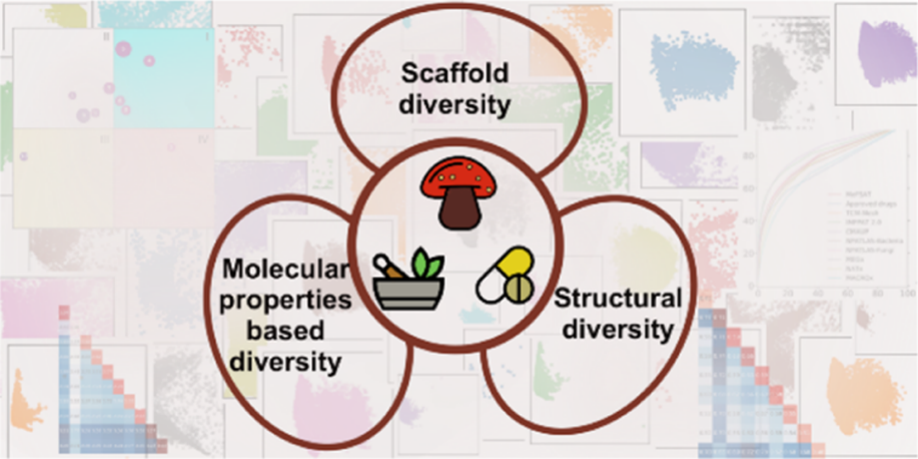

Medicinal fungi, including mushrooms, have well-documented therapeutic uses. In this study, we perform a cheminformatics-based investigation of the scaffold and structural diversity of the secondary metabolite space of medicinal fungi and, moreover, perform a detailed comparison with approved drugs, other natural product libraries, and semi-synthetic libraries. We find that the secondary metabolite space of medicinal fungi has similar or higher scaffold diversity in comparison to other natural product libraries analyzed here. Notably, 94% of the scaffolds in the secondary metabolite space of medicinal fungi are not present in the approved drugs. Further, we find that the secondary metabolites, on the one hand, are structurally far from the approved drugs, while, on the other hand, they are close in terms of molecular properties to the approved drugs. Lastly, chemical space visualization using dimensionality reduction methods showed that the secondary metabolite space has minimal overlap with the approved drug space. In a nutshell, our results underscore that the secondary metabolite space of medicinal fungi is a valuable resource for identifying potential lead molecules for natural product-based drug discovery.

## Introduction

Natural products, semi-synthetics, and synthetic libraries of different sources are being leveraged in high-throughput screening (HTS) to identify new antiviral, antibacterial, and anticancer agents.^1,2^ In addition, there is an increased focus toward natural product libraries for identification of new chemical entities with immunomodulatory, anti-aging, and cognitive enhancement properties to prevent diseases and promote holistic well-being.^3−5^ In this regard, the selection of appropriate chemical libraries with high diversity is a critical step in the drug discovery pipeline. Notably, chemical libraries with high structural diversity have a higher hit identification rate in HTS than similarly sized libraries with low structural diversity.^6,7^ Therefore, it is imperative to assess the diversity encoded by natural product libraries, which are a promising source of diverse chemical scaffolds.

Natural products from plants, fungi, bacteria, and marine organisms are rich sources of biologically relevant small molecules.^8^ Specifically, the natural product space of medicinal plants and fungi is more likely to be enriched with therapeutic small molecules.^9,10^ Many fungal secondary metabolites have been approved as drugs to treat human ailments. A prominent example is penicillin, the first of the class of broad-spectrum β-lactam antibiotics to be used clinically. Another example is lovastatin which is the first statin approved for clinical use. Lovastatin, initially isolated from the fungus *Aspergillus terreus,* is a widely used drug to lower total serum cholesterol and low-density lipoprotein cholesterol.^11^ Also, derivatives of fungal secondary metabolites have been approved as drugs. One such example is fingolimod, an approved drug for multiple sclerosis, obtained by synthetically modifying the fungal metabolite myriocin.^12^ Thus, several databases of natural products of plant and microbial origins have been developed to facilitate the ongoing efforts in natural product-based drug discovery.^13−17^ Specifically, there have been several efforts to develop and analyze phytochemical libraries of medicinal plants used in traditional medicine, such as TCM-Mesh^13^ and IMPPAT.^14,17^ In contrast, though medicinal fungi which include a variety of mushrooms have also been used in traditional medicine since ancient times,^18^ the secondary metabolite space of medicinal fungi remains comparatively much less explored. To this end, we previously created MeFSAT, a curated natural product database compiling information on 184 medicinal fungi, a chemical library of 1830 secondary metabolites produced by medicinal fungi, and therapeutic uses of the medicinal fungi.^19^ This enables the analysis of the diversity encoded by the secondary metabolite space of medicinal fungi, which in turn will facilitate their use in drug discovery and wellness research.

Medina-Franco and colleagues have developed several methods for quantifying and visualizing the structural diversity of chemical libraries. Medina-Franco et al.^20^ were among the first to perform a systematic analysis of the scaffold diversity using cyclic system retrieval (CSR) curves and Shannon entropy (SE). Later, they developed the consensus diversity plot (CDP) to assess the global diversity of the chemical libraries.^21^ Subsequently, these methods have been extensively used to compare and assess the structural diversity of chemical libraries, including natural products.^22−27^ Previously, González-Medina et al.^24^ have also done a comparative analysis of the scaffold diversity of 223 fungal secondary metabolites with approved drugs and commercial libraries. They found that the fungal secondary metabolites are structurally diverse with unique scaffolds not found in other libraries analyzed by them. However, all the studies to date on the analysis of the diversity of the fungal secondary metabolites were limited by a small library (<300 chemicals) created specifically to identify anti-cancer leads.^24,28,29^ In other words, to the best of our knowledge, no scientific study has been performed to assess the scaffold diversity of a large secondary metabolite library specifically curated from medicinal fungi.

In this study, we therefore performed a systematic analysis of the scaffold diversity of a large chemical library (>1800 chemicals) of secondary metabolites of medicinal fungi. Moreover, we compared the secondary metabolite space of medicinal fungi (MeFSAT) with nine different chemical libraries, including natural products, approved drugs, and commercial semi-synthetic libraries (Table 1), using scaffold diversity, structural diversity based on MACCS key structural fingerprints, and diversity in terms of molecular properties. We also used CDP to assess the global diversity of the chemical libraries. Finally, we used generative topographic mapping (GTM) and principal component analysis (PCA) to visualize and compare the chemical space of MeFSAT and other chemical libraries considered here.

**Table 1:** List of Chemical Libraries Analyzed in This Study^a^

chemical library	description	number of unique chemicals	reference
MeFSAT	secondary metabolites of medicinal fungi	1829	Vivek-Ananth et al., 2021^19^
Approved drugs	approved drugs from DrugBank	2466	Wishart et al., 2017^30^
TCM-Mesh	phytochemicals of Chinese herbs	10,127	Zhang et al., 2017^13^
IMPPAT 2.0	phytochemicals of Indian medicinal plants	17,915	Vivek-Ananth et al., 2022^17^
CMAUP	phytochemicals of medicinal and edible plants across the globe	47,187	Zeng et al., 2019^16^
NPATLAS-Bacteria	natural products in NPATLAS of bacterial origin	12,505	van Santen et al., 2019^15^
NPATLAS-Fungi	natural products in NPATLAS of fungal origin	19,966	van Santen et al., 2019^15^
MEGx	natural product library from a commercial vendor	6458	AnalytiCon Discovery^31^
NATx	semi-synthetic library from a commercial vendor	33,000	AnalytiCon Discovery^31^
MACROx	semi-synthetic library from a commercial vendor	4306	AnalytiCon Discovery^31^

a For each chemical library, the number of unique chemicals and the literature reference are provided.

## Results and Discussion

### Molecular Scaffolds of the Secondary Metabolite Space of Medicinal Fungi

MeFSAT^19^ is a dedicated resource compiling secondary metabolites produced by medicinal fungi. After building the manually curated MeFSAT^19^ database, we had performed a detailed analysis of the chemical space captured therein. Characterization of the molecular scaffolds in a chemical library enables identification of compounds with novel scaffolds that can be considered in the drug discovery pipeline. Previously, we had not computed the molecular scaffolds for the secondary metabolites in the MeFSAT^19^ database. In this study, we therefore identified the molecular scaffolds for the secondary metabolites of medicinal fungi (Methods).

Next, we updated the MeFSAT database by including the valuable information on molecular scaffolds identified in each secondary metabolite at three different levels, namely, G/N/B, G/N, and Graph, following the definition by Lipkus et al.^32,33^ (Methods). The updated database is openly accessible,^34^ and the users can filter secondary metabolites by selecting scaffolds of interest via the “Scaffold filter” tab under “Advanced Search” option (Figure S1). Moreover, the detailed information page for each secondary metabolite in the updated database now displays the identified scaffolds at the three levels (Figure S1). Also, to further facilitate the use of the MeFSAT database for drug discovery, we updated the secondary metabolite annotation with links to external databases which provide information on the commercial availability of the physical samples of the chemicals.^35,36^

**Figure 1:**
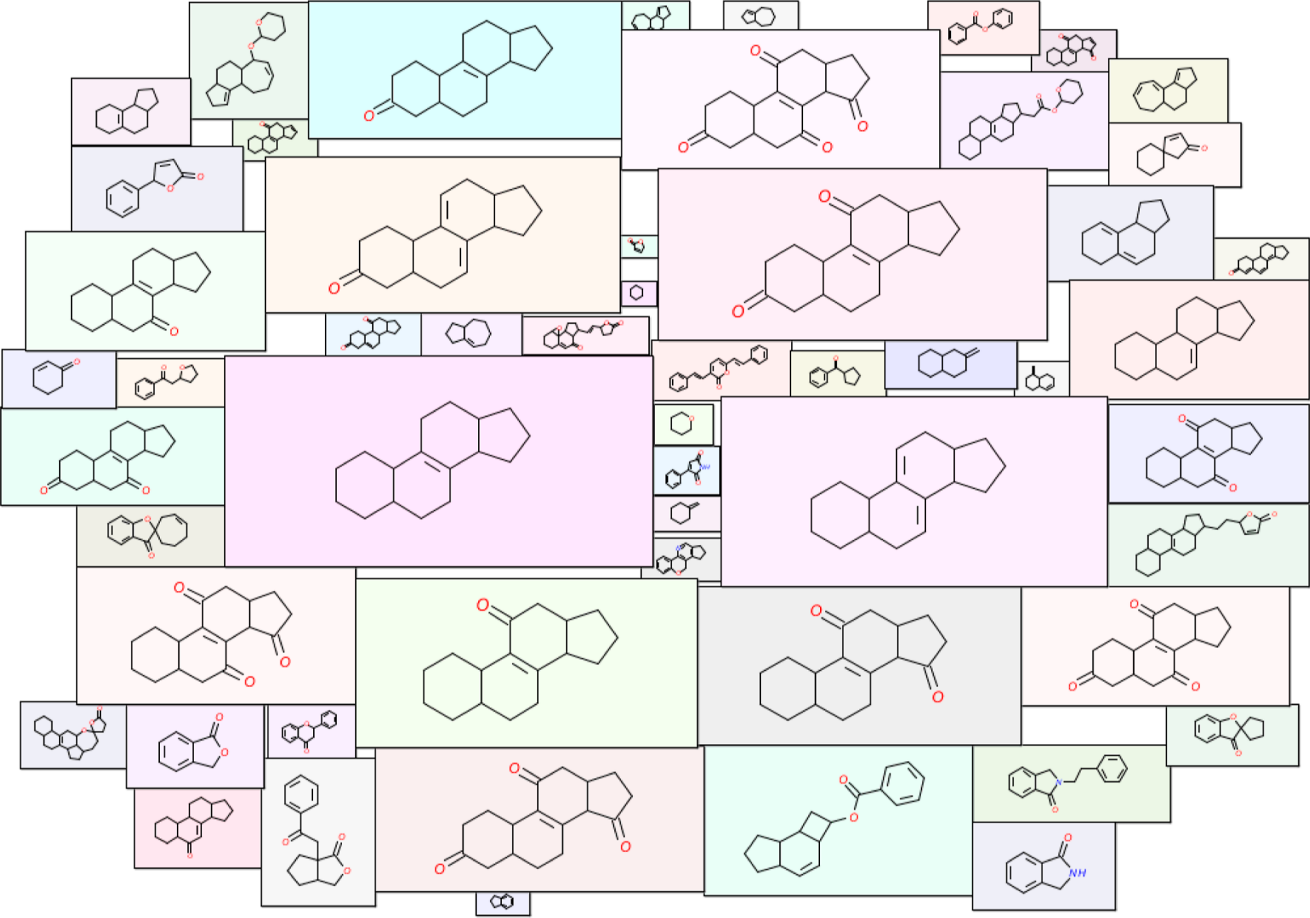
Molecular cloud visualization of the top scaffolds that occur in at least five secondary metabolites in MeFSAT. In this figure, the size of a scaffold image reflects its frequency of occurrence in the secondary metabolite space of medicinal fungi. Further, we considered only the cyclic chemicals while selecting the top scaffolds. Moreover, the benzene ring scaffold is omitted from this visualization as it is the most frequent scaffold in any large chemical library.

**Figure 2:**
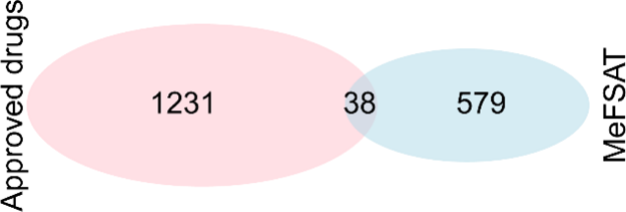
Venn diagram displays the overlap between the molecular scaffolds occurring in the secondary metabolite space of MeFSAT and approved drugs in DrugBank.

Overall, in the secondary metabolites of medicinal fungi obtained from MeFSAT, we found 618 unique scaffolds at the G/N/B level, including the pseudo-scaffold used to account for acyclic chemicals in the library (Table 2; Methods). Of these 618 scaffolds, 56 scaffolds occur in 5 or more secondary metabolites, and Figure 1 is a molecular cloud visualization^37,38^ of these frequent scaffolds after excluding the benzene ring scaffold. After computing the molecular scaffolds for the approved drug space compiled in DrugBank version 5.1.9,^30^ we found that there is minimal overlap between scaffolds in the secondary metabolites of medicinal fungi and scaffolds in approved drugs. 94% of the scaffolds identified in the secondary metabolites of medicinal fungi are not present in approved drugs (Figure 2). This result highlights the unique scaffolds present in the secondary metabolite space of medicinal fungi and therefore the potential of this natural product space for future drug discovery.

**Table 2:** Comparative Analysis of the Scaffold Diversity of the Secondary Metabolites in MeFSAT with Other Chemical Libraries^a^

chemical library	*M*	*N*	*N*_sing_	*N*/*M*	*N*_sing_/*M*	*N*_sing_/*N*	AUC	*P*_50_
MeFSAT	1829	618	370	0.338	0.202	0.599	0.786	7.443
Approved drugs	2466	1270	1026	0.515	0.416	0.808	0.729	11.102
TCM-Mesh	10,127	3949	2629	0.39	0.26	0.666	0.77	8.787
IMPPAT 2.0	17,915	5184	3344	0.289	0.187	0.645	0.824	3.492
CMAUP	47,187	11,118	6181	0.236	0.131	0.556	0.837	3.913
NPATLAS-Bacteria	12,505	4234	2463	0.339	0.197	0.582	0.78	9.258
NPATLAS-Fungi	19,966	6414	3779	0.321	0.189	0.589	0.794	7.141
MEGx	6458	2566	1723	0.397	0.267	0.671	0.767	9.08
NATx	33,000	11,445	6370	0.347	0.193	0.557	0.764	11.769
MACROx	4306	2039	1329	0.474	0.309	0.652	0.719	16.037

a Here, *M* is the size of the library, *N* is the total number of scaffolds (including the pseudo-scaffold for acyclic chemicals) in the library, *N*_sing_ is the total number of singleton scaffolds in the library, AUC is the area under the curve for the corresponding CSR curve, and *P*_50_ is the percentage of scaffolds required to retrieve 50% of chemicals in the library.

### Comparative Analysis of the Scaffold Diversity of Secondary Metabolite Space of Medicinal Fungi with Other Chemical Libraries

In this study, we compared the scaffold diversity of secondary metabolites of medicinal fungi (MeFSAT) with 9 other chemical libraries (Table 1; Methods). Table 2 provides the statistics on the number of scaffolds (*N*), the fraction of scaffolds per molecule (*N*/*M*), and the number of singleton scaffolds (*N*_sing_) for the 10 chemical libraries analyzed here.

In terms of the fraction of scaffolds per molecule, the secondary metabolite space of MeFSAT (*N*/*M* = 0.338) is similar to the libraries of natural products from fungi (NPATLAS-Fungi; *N*/*M* = 0.321) and natural products from bacteria (NPATLAS-Bacteria; *N*/*M* = 0.339). Although the library of approved drugs from DrugBank and the semi-synthetic library MACROx are among the smallest in terms of library size, the two chemical libraries were found to have a higher *N*/*M* ratio of 0.515 and 0.474, respectively. In terms of the fraction of singleton scaffolds per molecule, the secondary metabolite space of MeFSAT (*N*_sing_/*M* = 0.202) was found to have a higher value in comparison to relatively larger natural product libraries, namely, IMPPAT 2.0, CMAUP, and NPATLAS-Fungi, analyzed here (Table 2). Overall, in terms of the fraction of scaffolds per molecule and the fraction of singleton scaffolds per molecule, the secondary metabolite space of MeFSAT has scaffold diversity similar or higher in comparison to other natural product libraries analyzed here (Table 2).

### Analysis of Scaffold Diversity via Cyclic System Retrieval Curves

**Figure 3:**
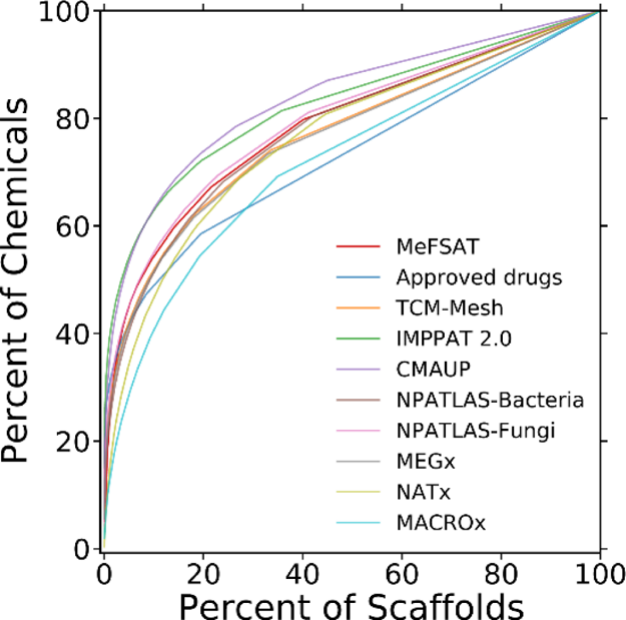
CSR curves for 10 different chemical libraries considered in this study. Note that a CSR curve close to the diagonal line indicates high scaffold diversity. The two metrics, namely, the AUC and the percentage of scaffolds required to retrieve 50% of chemicals (*P*_50_), derived from the CSR curves, also enable quantitative comparison of the scaffold diversity between chemical libraries.

Inspired by previous investigations,^17,20,25,39^ we computed CSR curves to quantify and compare the scaffold diversity of chemical libraries (Figure 3; Methods). From the CSR curves shown in Figure 3, it can be seen that the secondary metabolite space of MeFSAT has higher scaffold diversity in comparison to the larger natural product libraries IMPPAT 2.0 and CMAUP. Further, from the CSR curves shown in Figure 3, we find that the scaffold diversity of the secondary metabolite space of MeFSAT is similar to that of the natural product libraries NPATLAS-Fungi, NPATLAS-Bacteria, TCM-Mesh, and MEGx. On the other hand, we find that the approved drugs from DrugBank and the semi-synthetic library MACROx have the highest scaffold diversity among the chemical libraries analyzed here.

Moreover, we performed a quantitative comparison of the different chemical libraries using two metrics derived from the CSR plot, namely, area under the curve (AUC) and percentage of scaffolds required to retrieve 50% of chemicals (*P*_50_) (Methods). As mentioned in the Methods section, a lower AUC value and a higher *P*_50_ value are indicators of higher scaffold diversity. Table 2 lists the two metrics computed from the CSR curves shown in Figure 3 for different chemical libraries analyzed here. We find that the secondary metabolite space of MeFSAT has an AUC value similar to other natural product libraries. Interestingly, we also find that the *P*_50_ values distinguish on the one hand the semi-synthetic libraries, NATx, and MACROx, and on the other hand the approved drugs from the natural product libraries analyzed here (Table 2).

### Distribution of Chemicals across the Most Populated Scaffolds in Different Libraries

We computed the scaled Shannon entropy (SSE) for each chemical library to quantify the nature of distribution of chemicals across the topmost populated scaffolds (Methods). The maximum value (1) of SSE indicates an even distribution of the chemicals across the topmost populated scaffolds whereas the minimum value (0) of SSE indicates that all chemicals have the same scaffold. In Table 3, we present the computed SSE values by considering the top 5 (SSE5) to top 70 (SSE70) most populated scaffolds for each chemical library analyzed here. The secondary metabolite space of MeFSAT (SSE values: 0.979 to 0.876) has the highest diversity among all the natural product libraries analyzed here. The semi-synthetic libraries NATx (SSE values: 0.994 to 0.984) and MACROx (SSE values: 0.940 to 0.957) have the highest SSE values among the libraries considered here, and moreover, the SSE values are closer to 1 for the two libraries, indicating high scaffold diversity. Note that the scaffold diversity interpreted from SSE values is based only on the topmost populated scaffolds, whereas the AUC based on CSR curves is based on analysis of all the scaffolds in a chemical library, and therefore, SSE and AUC measure different aspects of the diversity. This explains the reason behind the approved drugs having the lowest SSE values (0.675 to 0.680) in spite of having a low AUC value computed from the CSR curve (Figure 3).

**Table 3:** SSE Computed Using the Most Populated Scaffolds for the Chemical Libraries Analyzed in This Study^a^

chemical library	SSE5	SSE10	SSE20	SSE30	SSE40	SSE50	SSE60	SSE70
MeFSAT	0.979	0.956	0.929	0.913	0.899	0.888	0.882	0.876
Approved drugs	0.675	0.618	0.626	0.64	0.654	0.663	0.672	0.68
TCM-Mesh	0.812	0.782	0.787	0.794	0.799	0.803	0.805	0.807
IMPPAT 2.0	0.671	0.649	0.663	0.669	0.678	0.685	0.688	0.691
CMAUP	0.785	0.766	0.781	0.781	0.784	0.788	0.792	0.796
NPATLAS-Bacteria	0.79	0.778	0.784	0.795	0.805	0.813	0.82	0.827
NPATLAS-Fungi	0.849	0.856	0.863	0.866	0.867	0.867	0.867	0.867
MEGx	0.868	0.857	0.851	0.855	0.857	0.859	0.859	0.859
NATx	0.994	0.991	0.986	0.985	0.985	0.985	0.984	0.984
MACROx	0.94	0.95	0.952	0.953	0.955	0.956	0.957	0.957

a The table provides the computed SSE values for the 5 most populated scaffolds (SSE5) to the computed SSE values for the 70 most populated scaffolds (SSE70) for different chemical libraries.

**Figure 4:**
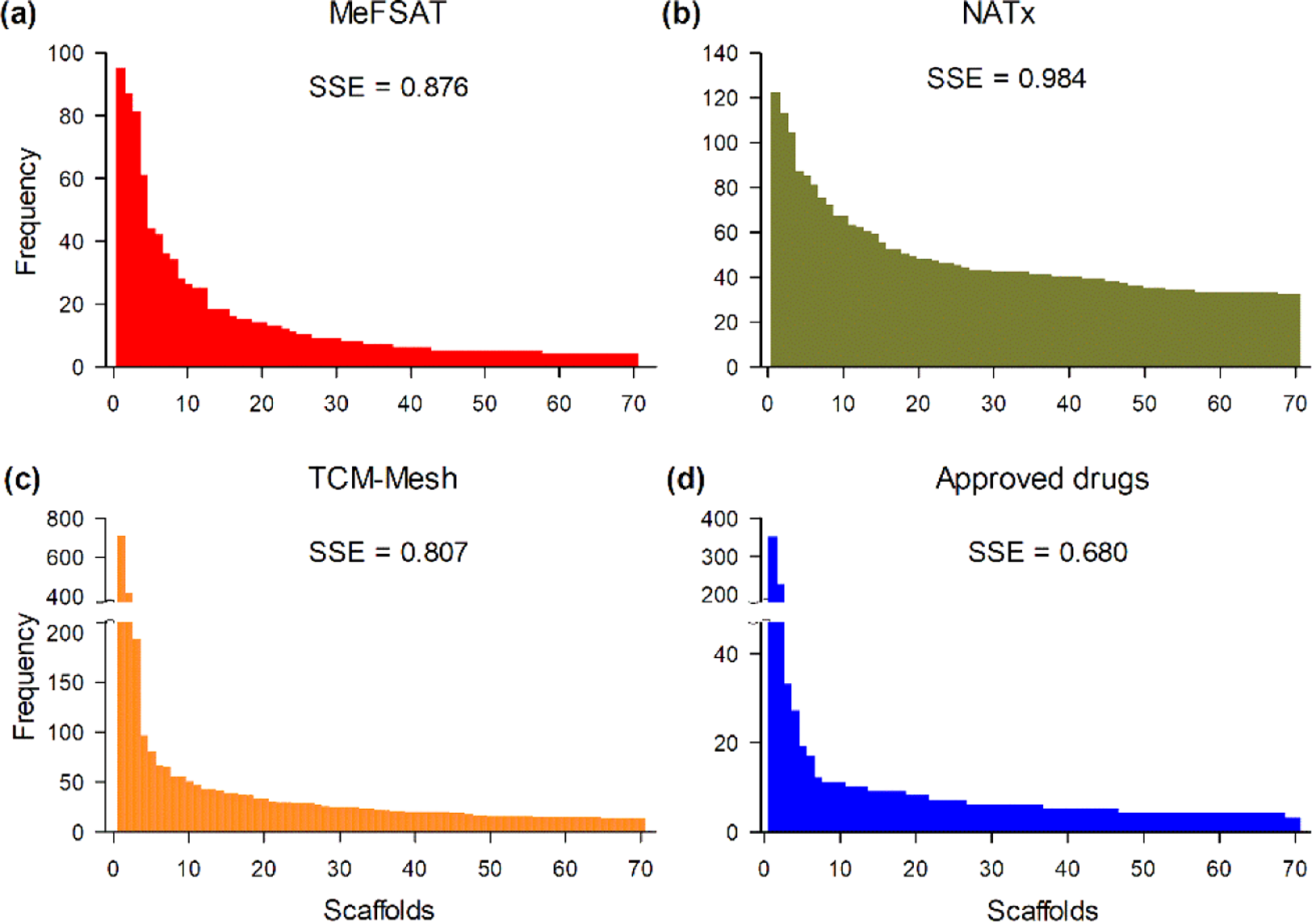
Distribution of chemicals across the top 70 most populated scaffolds in: (a) secondary metabolites in MeFSAT, (b) semi-synthetic library NATx, (c) phytochemical library TCM-Mesh, and (d) Approved drugs.

Figure 4 displays the distribution of the number of chemicals across the top 70 most populated scaffolds in MeFSAT, the semi-synthetic library NATx, the phytochemical library TCM-Mesh, and the approved drugs. The corresponding distributions for other libraries analyzed here are shown in Figure S2. Libraries with a low SSE70 value have a less even distribution of chemicals, as can be seen in the case of approved drugs (Figure 4d). In contrast, the semi-synthetic library NATx, which has the highest SSE70 value, has a more even distribution of chemicals (Figure 4b).

### Inter- and Intra-Library Distance between the Secondary Metabolite Space of Medicinal Fungi and Other Chemical Libraries

**Figure 5:**
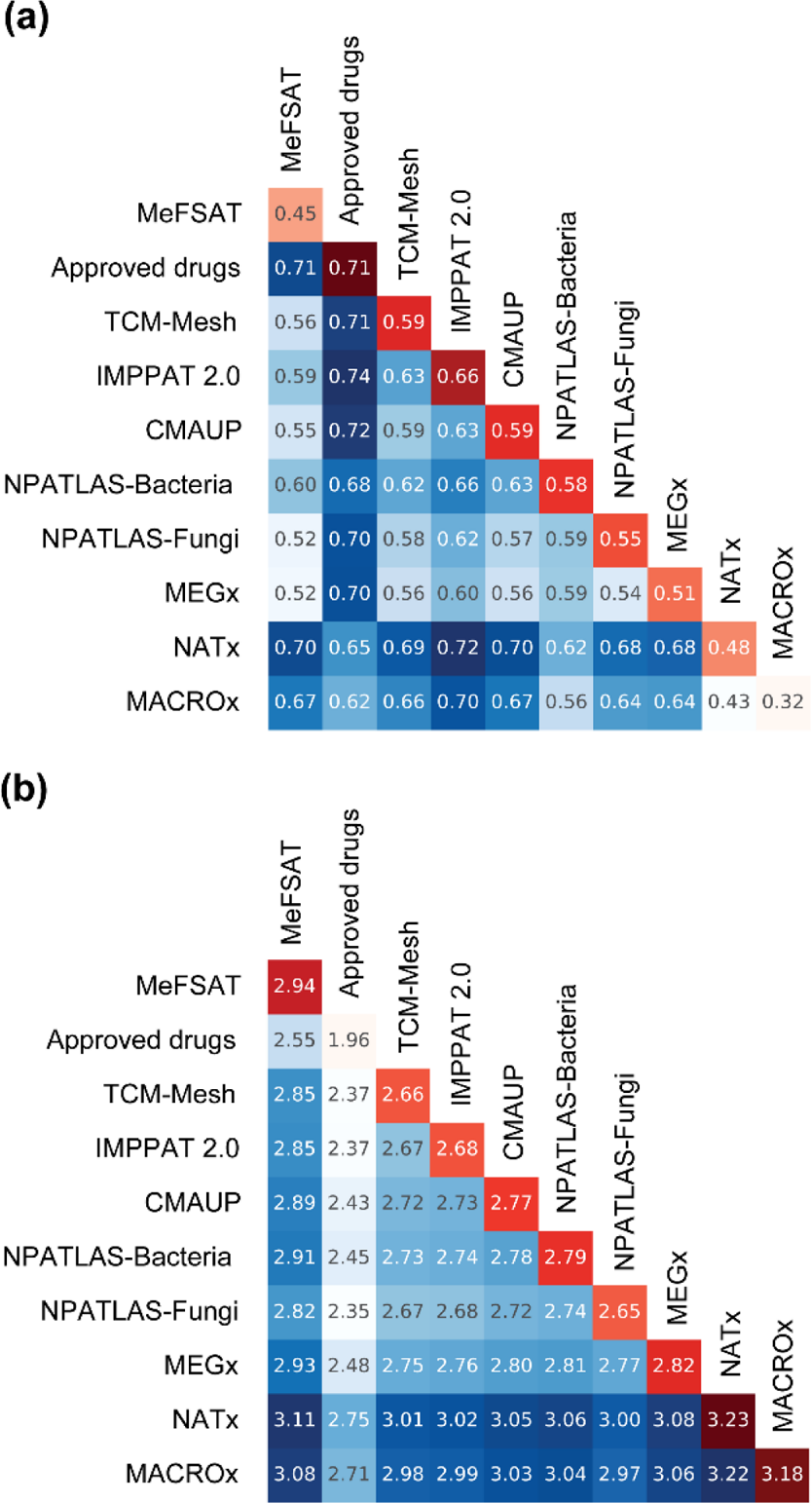
THPs for the chemical libraries analyzed here. (a) THP based on Soergel distance using MACCS key fingerprints and (b) THP based on Euclidean distance of molecular properties. The off-diagonal cells show the inter-library distance and are colored in gradients of blue. Dark blue indicates low similarity and light blue indicates high similarity between libraries. The diagonal cells show the intra-library diversity and are colored in gradients of red. Dark red indicates high diversity and light red indicates low diversity within the library.

By employing the Soergel distance using MACCS keys fingerprints and the Euclidean distance using six molecular properties, we quantified the inter- and intra-library distances for the chemical libraries analyzed here (Methods). Figure 5a,b display the triangular heatmap plots (THPs) summarizing the inter- and intra-library distances for the chemical libraries based on: (a) the Soergel distance computed using MACCS keys fingerprints, and (b) the Euclidean distance computed using molecular properties, respectively. In Figure 5, the diagonal cells of THPs show the intra-library distance colored in gradients of red, wherein darker shades of red indicate high diversity and lighter shades of red indicate low diversity. Moreover, the off-diagonal cells in THPs show the inter-library distances colored in gradients of blue, wherein darker shades of blue indicate high inter-library distance (i.e., low similarity between the pair of libraries) and lighter shades of blue indicate low inter-library distance (i.e., high similarity between the pair of libraries).

#### Structural Diversity Based on Soergel Distance Using MACCS Key Fingerprints

From the off-diagonal cells in THP based on structural fingerprints shown in Figure 5a, it is evident that secondary metabolites in MeFSAT are similar to those in other natural product libraries analyzed here. In particular, the secondary metabolite space of MeFSAT is closest to NPATLAS-Fungi (0.52) and MEGx (0.52). In contrast, the secondary metabolite space of MeFSAT is farthest from the approved drugs (0.71), followed by semi-synthetic libraries, NATx (0.70) and MACROx (0.67). Notably, the high inter-library distance between MeFSAT and approved drugs highlights that the MeFSAT library is more suitable for HTS to identify new chemical entities. From the diagonal cells in THP based on structural fingerprints shown in Figure 5a, we observe that MeFSAT has an intermediate intra-library distance (0.45), whereas the approved drug space has the highest intra-library distance (0.71), followed by the IMPPAT 2.0 phytochemical space (0.66).

#### Chemical Diversity Based on Euclidean Distance Using Molecular Properties

From the off-diagonal cells in THP based on molecular properties shown in Figure 5b, it is observed that the secondary metabolites in MeFSAT are more similar to natural product libraries and approved drugs, while the secondary metabolites in MeFSAT are less similar to the semi-synthetic libraries, NATx and MACROx. In particular, the secondary metabolites in MeFSAT are closest to the NPATLAS-Fungi (2.82) based on molecular properties. Interestingly, the secondary metabolite space of MeFSAT is found to be similar to the space of approved drugs based on the molecular properties, in spite of the high inter-library distance based on structural fingerprints (Figure 5a) and minimal scaffold overlap between the two libraries (Figure 2). This observation highlights that the MeFSAT library is enriched with secondary metabolites with favorable molecular properties similar to approved drugs though being structurally diverse from the approved drugs, and this makes them more suitable for HTS to identify new chemical entities. From the diagonal cells in THP based on molecular properties shown in Figure 5b, it is seen that MeFSAT has the highest intra-library distance (2.94) among the natural product libraries considered here, while the semi-synthetic libraries, NATx (3.23) and MACROx (3.18), have the highest intra-library distance across all the libraries analyzed here. Also, when comparing the structural fingerprint-based intra-library distance (Figure 5a) and the molecular properties-based intra-library distance (Figure 5b), the approved drugs were found to have the highest diversity based on structural fingerprints but low diversity based on molecular properties. This contrasting observation can be understood by the fact that the drug development pipeline is often constrained by the physicochemical properties, which limit the diversity of the molecular properties of the approved drugs.^40,41^

### Global Diversity Analysis with Consensus Diversity Plot

**Figure 6:**
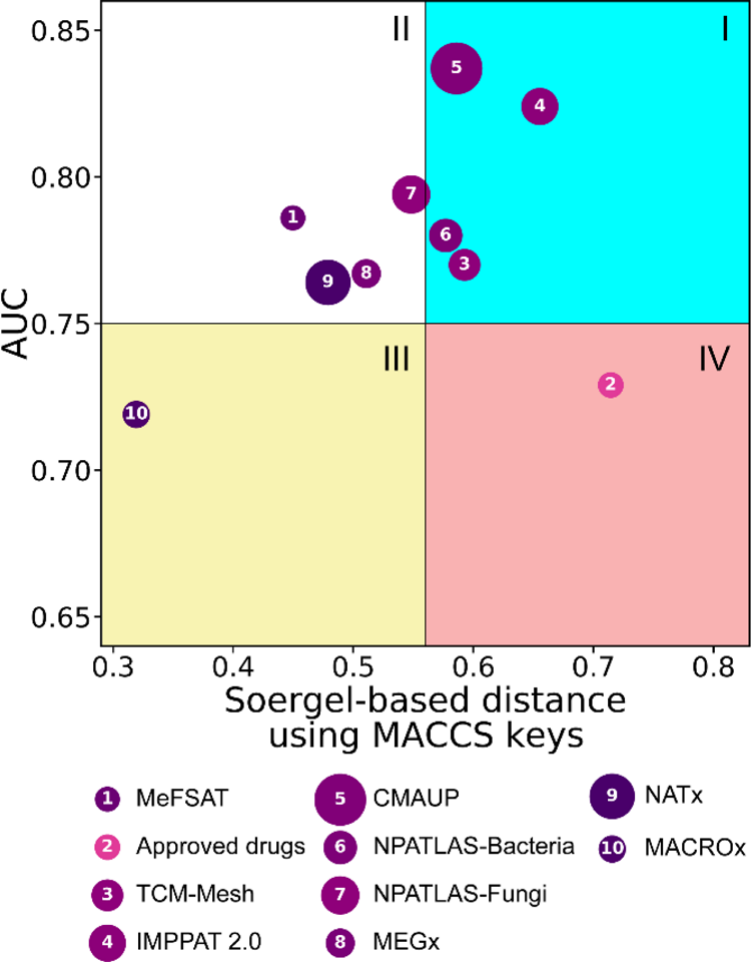
CDP visualizing the global diversity of the chemical libraries. The *x*-axis represents the Soergel-based distance using MACCS keys and the *y*-axis represents the AUC from the CSR curve. The CDP is divided into four quadrants: I in cyan, II in white, III in yellow, and IV in salmon-red. The data points are colored in a pink to purple gradient, with light pink indicating low diversity and dark purple indicating high diversity based on molecular properties. The relative size of the chemical libraries is reflected in the size of the data points.

Figure 6 shows the CDP which captures the global diversity of the chemical libraries analyzed here (Methods). Briefly, in the CDP, the *x*-axis gives the Soergel-based intra-library distance computed using MACCS keys fingerprints, the *y*-axis gives the AUC from the CSR curves, the color of the data points captures the molecular properties-based intra-library distance computed using the Euclidean distance function, and the relative size of the chemical libraries is reflected in the size of the data points (Methods). Furthermore, the data points (corresponding to different chemical libraries) fall in either one of the four quadrants of the CDP. In a CDP plot, the chemical libraries in quadrant IV (salmon-red) are more diverse based on both scaffold and structural fingerprints, the libraries in quadrant III (yellow) have high scaffold diversity, the libraries in quadrant I (cyan) have high structural diversity, and the libraries in quadrant II (white) have relatively lower diversity (Figure 6).

From Figure 6, we find that secondary metabolites in MeFSAT have higher scaffold diversity compared to larger natural product libraries such as CMAUP, IMPPAT 2.0, and NPATLAS-Fungi analyzed here. Further, the secondary metabolites in MeFSAT have intermediate structural diversity similar to NPATLAS-Fungi, MEGx, and the semi-synthetic library NATx. Based on the color of the data points, we find that the secondary metabolite space of MeFSAT has a similar diversity in terms of molecular properties to other natural product libraries analyzed here. Moreover, we find that MeFSAT and NPATLAS-Fungi libraries are in the same quadrant of the CDP, and thus, the two libraries have similar global diversity even though the library size of NPATLAS-Fungi is ∼10-fold larger than MeFSAT (Table 1). As expected, the library of approved drugs falls in the quadrant IV, underscoring the high diversity of the approved drug space. We also find that the majority of the natural product libraries are in quadrant I and thus have high structural diversity.

By comparing the colors of the data points in Figure 6, we find that all the natural product libraries analyzed here have an intermediate diversity in terms of molecular properties, whereas the semi-synthetic libraries, NATx and MACROx, have a high diversity in terms of molecular properties. As can be seen in Figure 5b, we also find that the library of approved drugs has a lower diversity in terms of molecular properties. In sum, the CDP captures the global diversity of the chemical libraries, enabling combined visual interpretations of the several metrics computed in this investigation.

### Visualization of Chemical Spaces

**Figure 7:**
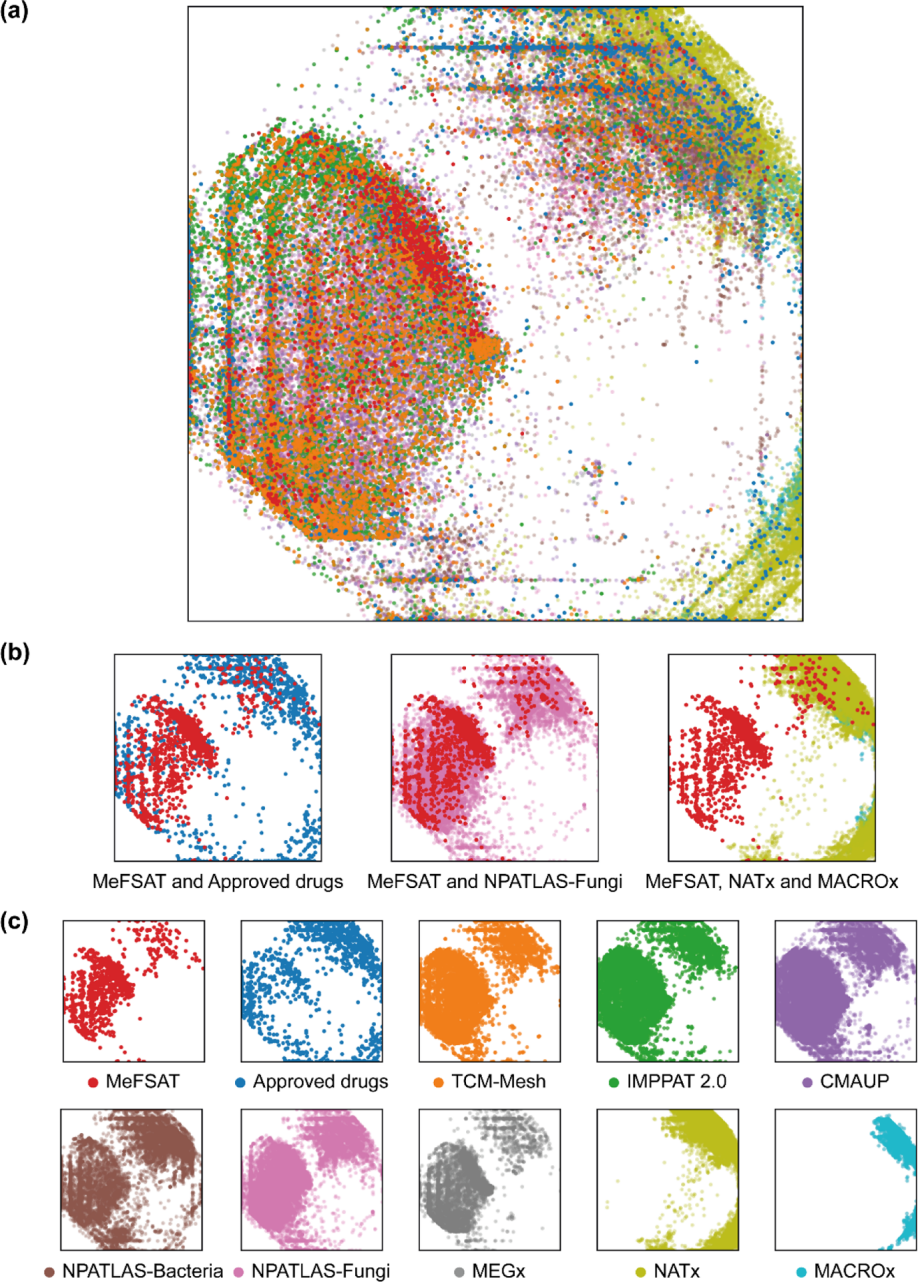
Visualization of the chemical spaces generated via GTM using MACCS key structural fingerprints for the libraries analyzed here. (a) Visualization of all chemical libraries analyzed here. (b) Visualization of MeFSAT and approved drugs, MeFSAT and NPATLAS-Fungi, and MeFSAT, NATx, and MACROx. (c) Visualization of each individual chemical library. The color used to represent each chemical library in the visualization is provided in (c) along with the corresponding library name.

Figure 7 is a visualization of the chemical spaces corresponding to the different libraries analyzed here, and the visualization was generated via GTM using MACCS keys structural fingerprints (Methods). The chemical space of the secondary metabolites in MeFSAT overlaps with the chemical space of other natural product libraries (Figure 7), and in particular, it is found to be similar to the chemical space of NPATLAS-Fungi as per expectation (Figure 7b). This finding also corroborates our similar observation from Figure 5a. The chemical space of the approved drugs was found to be more spread out with minimal overlap with MeFSAT (Figure 7b). This is in alignment with our previous findings that the secondary metabolite space of MeFSAT is structurally diverse from the space of approved drugs (Figures 2 and 5a). The chemical space of the semi-synthetic libraries, NATx and MACROx, was found to occupy a different region in the GTM-based visualization, which is underrepresented by the natural product libraries, including MeFSAT (Figure 7b).

Figure S3 displays the visualization of the chemical spaces generated via GTM using the six molecular properties for the different libraries analyzed here (Methods). The secondary metabolite space of MeFSAT is more spread out in comparison to the space of approved drugs (Figure S3). Also, the natural product libraries analyzed here occupy similar regions of the chemical space, wherein they occupy most regions in the two-dimensional visualization except for the regions closer to the left and bottom boundaries (Figure S3c). The semi-synthetic library NATx was found to be more spread out covering regions not occupied by the natural product libraries (Figure S3).

Figure S4 displays the visualization of the chemical spaces generated via PCA using MACCS keys structural fingerprints for the different libraries analyzed here (Methods). The observations on different chemical spaces analyzed here from Figure S4 generated via PCA closely follow those obtained from visualization generated via GTM using MACCS keys fingerprints. The visualization of the chemical spaces generated via PCA using six molecular properties (Figure S5) for the different libraries analyzed here was found to be less discriminative with the different libraries occupying a similar region in the lower-dimensional space.

## Conclusions

In the present investigation, we analyzed and compared the scaffold and structural diversity of the secondary metabolite space of medicinal fungi (as compiled in the MeFSAT database) with nine different chemical libraries, including natural products, approved drugs, and semi-synthetic libraries. We find that the secondary metabolite space of MeFSAT has equal or higher scaffold diversity in comparison to other natural product libraries (Tables 1 and 2; Figure 3). Also, we updated the MeFSAT database with the information on identified scaffolds in the secondary metabolites of medicinal fungi (Figure S1).

Apart from analyzing the scaffold diversity of the chemical libraries, we also analyzed the structural diversity and diversity in terms of molecular properties (Figure 5). Based on the structural diversity analysis, MeFSAT is found to be structurally closer to other natural product libraries and structurally farthest from the approved drugs and semi-synthetic libraries. In terms of molecular properties, MeFSAT is found to be closer to the natural product libraries and approved drugs, whereas it is farther from the semi-synthetic libraries. Interestingly, we also find that the MeFSAT library has minimal scaffold overlap with the approved drugs (Figure 2). This highlights the suitability of the MeFSAT library for HTS to identify new chemical entities.

From the global diversity analysis of the chemical libraries (Figure 6), we find that the MeFSAT library has intermediate structural diversity similar to natural product libraries such as NPATLAS-Fungi and MEGx, and the semi-synthetic library NATx, and has higher scaffold diversity in comparison to large-sized natural product libraries such as CMAUP and IMPPAT 2.0. Further, we find that the MeFSAT and NPATLAS-Fungi fall in the same quadrant of the CDP, and thus, they have similar global diversity (Figure 6). By visualizing the chemical spaces corresponding to the different chemical libraries, we find that the secondary metabolite space of MeFSAT is similar to other natural product libraries, and moreover, the secondary metabolite space of MeFSAT has minimal overlap with the approved drug space (Figure 7).

Lastly, one of the key findings of this study based on observations from multiple analyses is that the secondary metabolites of medicinal fungi (in MeFSAT) are scaffold-wise and structure-wise distant (dissimilar) from the approved drugs (Figures 2 and 5a). This observation alone cannot be used to infer that there are metabolites in fungi that can be used as drugs. Because consider a case where you have a library of chemicals each made of only nitrogen or oxygen atoms alone. Even in this case, the library will be structurally distant from the approved drugs, but the library will not be much use for drug discovery research. In this regard, the secondary metabolites of medicinal fungi, though scaffold-wise and structure-wise distant from the approved drugs, have molecular properties (that are important for drug-likeness) similar to the approved drugs (Figure 5b). This makes the secondary metabolites of medicinal fungi captured in MeFSAT more suitable for identifying novel drugs with hitherto unknown chemical scaffolds.

There are several challenges in the identification and development of drugs from fungal secondary metabolites, which include the availability of physical samples for conducting clinical studies, pharmacokinetics and pharmacodynamics of the secondary metabolites, and possible toxicity of the secondary metabolites. We believe the updated MeFSAT database and the results from our extensive analysis of the secondary metabolite space of medicinal fungi using molecular scaffolds, structural fingerprints, and molecular properties will facilitate the ongoing efforts to identify novel drugs from fungal secondary metabolites.

## Methods

### Compilation and Preprocessing of Chemical Libraries

For this comparative analysis, the list of secondary metabolites of medicinal fungi was obtained from our previously published database, Medicinal Fungi Secondary Metabolites And Therapeutics^19^ (MeFSAT). The chemical diversity of the secondary metabolite space of medicinal fungi was compared with the list of approved drugs compiled in DrugBank version 5.1.9,^30^ phytochemicals, microbial natural products, and commercial semi-synthetic libraries. Specifically, we considered the following phytochemical libraries, namely TCM-Mesh^13^ which compiles phytochemicals from Chinese herbs, IMPPAT 2.0^17^ which compiles phytochemicals from Indian medicinal plants, and CMAUP^16^ which compiles phytochemicals from medicinal and edible plants across the globe. Moreover, we subdivided the microbial natural product library, NPATLAS,^15^ for this analysis into chemicals of fungal origin (NPATLAS-Fungi) and chemicals of bacterial origin (NPATLAS-Bacteria). Though 1202 secondary metabolites of medicinal fungi captured in MeFSAT are also present in NPATLAS-Fungi, MeFSAT captures the secondary metabolite space specific to medicinal fungi, whereas the NPATLAS-Fungi captures a more generic secondary metabolite space of fungi. The large overlap between the MeFSAT and NPATLAS-Fungi libraries is not surprising because both capture the secondary metabolite space of fungi. Further, while compiling secondary metabolites in MeFSAT, we had made use of the NPATLAS database as one of the resources to retrieve chemical structures of secondary metabolites reported in published literature. Lastly, we also considered another natural product library, MEGx, and two semi-synthetic libraries namely, NATx and MACROx, from a commercial vendor.^31^Table 1 provides a summary of the different chemical libraries analyzed here. Notably, the chemical libraries in SDF file format were cleaned and deduplicated to create non-redundant lists using MayaChemTools.^42^ The compound overlap between the chemical libraries analyzed in this study is shown in Figure S6. Note that we used the chemical libraries as provided by the reference databases (Table 1), and the diversity analysis presented in this study does not take into consideration the stereochemistry of the chemicals.

### Computation of Molecular Scaffolds

The scaffolds capture the core molecular framework of a chemical, and this concept has been widely used to assess and compare the scaffold diversity of chemical libraries.^17,24,25,32,33,39^ In this study, we used the scaffold definition proposed by Bemis-Murcko^43^ to compute the molecular scaffolds of the chemicals in different libraries, wherein the scaffold is represented by all the ring systems and linkers connecting them. Based on this definition, only chemicals with cyclic systems have a scaffold. Since the analyzed libraries contain both cyclic and acyclic chemicals, the acyclic chemicals have been assigned a pseudo-scaffold in this work.

Following Lipkus et al.,^32,33^ one can compute the molecular scaffolds in different chemical libraries at three different levels, namely, graph/node/bond (G/N/B) level, graph/node (G/N) level, or graph level (Figure S7). The scaffold at G/N/B level has connectivity, element and bond information, and thus making it more informative than G/N or graph level. Hence, we analyzed the scaffold diversity of different libraries using molecular scaffolds computed at the G/N/B level for each chemical in this study. The scaffold computations were performed using custom in-house Python scripts employing RDKit.^44^

### Quantifying the Scaffold Diversity

Previous investigations^17,20,24,25,32,33,39^ have shown that CSR plots help in quantifying the scaffold diversity of chemical libraries. Using the scaffold information at the G/N/B level, we plotted the CSR curves for each chemical library considered here. In a CSR curve for a chemical library, the percentage of scaffolds is plotted on the *x*-axis, and the percentage of compounds containing those scaffolds is plotted on the *y*-axis. From the CSR curves, we computed two metrics, namely, the AUC and the percentage of scaffolds required to retrieve 50% of the chemicals (*P*_50_), to quantify and compare the scaffold diversity of the different chemical libraries. The scaffold diversity of a chemical library has a maximum value when the corresponding CSR curve is a diagonal line, which implies that 50% of scaffolds will retrieve 50% of the compounds in the library (*P*_50_) and the AUC value is 0.5.

The SE is employed to characterize the distribution of chemicals among the most populated scaffolds^20,45^ in a chemical library. For a selected population of *P* chemicals and top *n* scaffolds in a library, SE is defined as
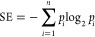
1where
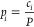
2

In the above equations, *c*_*i*_ is the number of chemicals containing the scaffold *i*, and *p*_*i*_ is the probability of the occurrence of the scaffold *i* in *P* chemicals containing a total of *n* scaffolds. The maximum possible value of SE is log_2_*n,* wherein all the *P* chemicals are evenly distributed among *n* scaffolds, and this represents high scaffold diversity in the library. The minimum possible value of SE is 0, wherein all the *P* chemicals have the same scaffold, and this represents low scaffold diversity in the library. Since SE is dependent upon the number of scaffolds *n*, we scaled SE by dividing it with the maximum value of SE. The SSE is defined as
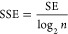
3

It is evident that SSE can take values from 0 to 1, where 0 corresponds to low scaffold diversity and 1 corresponds to high scaffold diversity of the chemical library.

### Inter- and Intra-Library Distance Based on Structural Fingerprints and Molecular Properties

We quantified the inter- and intra-library distances between the different chemical libraries using structural fingerprints and molecular properties of the chemicals. We computed the Molecular ACCess System (MACCS) keys fingerprints with 166 bits for each chemical using RDKit.^44^ To compare the similarity between two libraries, we computed the Soergel distance, which is a complement of the Tanimoto coefficient, using the binary fingerprints of chemical structures.^46^ If *x* and *y* are the binary fingerprints for two chemicals, then the corresponding Soergel distance can be computed as follows
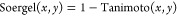
4where
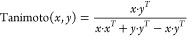
5

We computed the Tanimoto coefficient for a pair of chemicals using RDKit.^44^ The similarity coefficient of chemicals across two libraries, *D*_*u*_ and *D*_*v*_, that is, inter-library distance, was computed using Soergel-based inter-library distance *d*_*uv*_ following Owen et al.^46^ and is given by
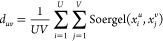
6

In the above equation, *U* and *V* are the number of chemicals in the two libraries *D*_*u*_ and *D*_*v*_. The diversity of chemicals in a single library or intra-library distance can be computed by modifying eq 6 and is given by
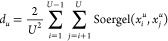
7

Further, we computed six molecular properties important for drug-likeness^47−49^ namely, hydrogen bond donors (HBD), hydrogen bond acceptors (HBA), octanol/water partition coefficient (LogP), molecular weight (MW), topological polar surface area (TPSA), and number of rotatable bonds (RTB), for each chemical using RDKit.^44^ Notably, these molecular properties were previously employed to compare chemical diversity across different libraries.^24^ The inter-library distance based on the six molecular properties between two libraries *D*_*u*_ and *D*_*v*_ containing *U* and *V* chemicals, respectively, was computed by measuring the Euclidean distance function^50^ and is given by
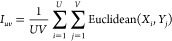
8where the  is given as
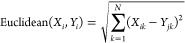
9

In the above equations, *X*_*i*_ and *Y*_*j*_ represent *N*-dimensional vectors containing molecular properties of chemicals *i* and *j* in libraries *D*_*u*_ and *D*_*v*_, respectively.

### Consensus Diversity Plots

CDP is a two-dimensional visualization used to compare the diversity of chemical libraries.^21^ CDP captures four important properties to characterize the diversity of the chemical libraries. First, the structural fingerprint-based diversity of a library, captured by Soergel-based intra-library distance using MACCS keys fingerprints, is plotted on the *x*-axis of CDP. Second, the scaffold diversity of a library, captured by AUC from the corresponding CSR curve, is plotted on the *y*-axis of CDP. Third, the data points in CDP are colored using a pink-to-purple gradient to capture the molecular properties based intra-library distance computed using the Euclidean distance function. Fourth, the relative size of the chemical libraries is represented by the size of the data points in CDP. Following González-Medina et al.*,*^21,24^ we analyzed the CDP by partitioning it into 4 quadrants which are differentiated by distinct colors. To define the four quadrants in CDP, we considered the median of the Soergel-based intra-library distance and an AUC value of 0.75 to assign the thresholds for *x*-axis and *y*-axis, respectively.

### Visualization of Chemical Spaces

In cheminformatics literature,^46,51^ multiple methods have been proposed for dimensionality reduction and visualization of chemical spaces. Of these methods, generative topographic mapping^52^ (GTM) and principal component analysis^53^ (PCA) have been widely used for chemical space visualization. Using GTM and PCA, we visualized different chemical libraries based on MACCS keys fingerprints and six molecular properties important for drug-likeness. PCA projects the high-dimensional data to a low-dimensional space using linear mapping.^53^ Although PCA is widely used for dimensionality reduction, it is unsuitable for nonlinear data.^54^ In contrast, GTM is a nonlinear method that projects the high-dimensional data to a two-dimensional space using radial basis function.^52^

To represent any chemical space using structural fingerprints, we employed MACCS keys fingerprints with 166 binary bits that capture the presence or absence of structural features in a chemical structure. To represent any chemical space using molecular properties, we employed the six molecular properties, namely HBD, HBA, LogP, MW, TPSA, and RTB, as described in the preceding section. The high-dimensional input data for a chemical library in terms of either structural fingerprints or molecular properties was then mapped to a two-dimensional space using: (a) GTM implemented using ugtm^55^ Python package and (b) PCA implemented using scikit-learn^56^ Python package. Subsequently, the dataset corresponding to a chemical library after dimensionality reduction is visualized using Matplotlib^57^ Python package.

## Supporting Information Available

Screenshots of the Scaffold filter tab under the Advanced Search option and the detailed information page for a secondary metabolite in the updated MeFSAT database; distribution of chemicals across the top 70 most populated scaffolds in MACROx, NPATLAS-Fungi, MEGx, NPATLAS-Bacteria, CMAUP, and IMPPAT 2.0 libraries; visualization of the chemical spaces generated via GTM using molecular properties; PCA using MACCS key structural fingerprints; PCA using molecular properties for the libraries analyzed in this study; compound overlap between the chemical libraries analyzed in this study; and molecular scaffold of a secondary metabolite at three different levels (PDF)

## References

[ref1] Mayr L. M.; Bojanic D. Novel Trends in High-Throughput Screening. Curr. Opin. Pharmacol. 2009, 9, 580–588. 10.1016/j.coph.2009.08.004. 19775937

[ref2] Abel U.; Koch C.; Speitling M.; Hansske F. G. Modern Methods to Produce Natural-Product Libraries. Curr. Opin. Chem. Biol. 2002, 6, 453–458. 10.1016/s1367-5931(02)00338-1. 12133720

[ref3] Appendino G.; Minassi A.; Taglialatela-Scafati O. Recreational Drug Discovery: Natural Products as Lead Structures for the Synthesis of Smart Drugs. Nat. Prod. Rep. 2014, 31, 880–904. 10.1039/c4np00010b. 24823967

[ref4] Ding A.-J.; Zheng S.-Q.; Huang X.-B.; Xing T.-K.; Wu G.-S.; Sun H.-Y.; Qi S.-H.; Luo H.-R. Current Perspective in the Discovery of Anti-Aging Agents from Natural Products. Nat. Prod. Bioprospect. 2017, 7, 335–404. 10.1007/s13659-017-0135-9. 28567542 PMC5655361

[ref5] Haddad P. S.; Azar G. A.; Groom S.; Boivin M. Natural Health Products, Modulation of Immune Function and Prevention of Chronic Diseases. Evidence-Based Complementary Altern. Med. 2005, 2, 513 10.1093/ecam/neh125. PMC1297498 16322809

[ref6] Van Drie J. H.; Lajiness M. S. Approaches to Virtual Library Design. Drug Discovery Today 1998, 3, 274–283. 10.1016/s1359-6446(98)01186-6.

[ref7] Harper G.; Pickett S.; Green D. Design of a Compound Screening Collection for Use in High Throughput Screening. Comb. Chem. High Throughput Screening 2004, 7, 63–70. 10.2174/138620704772884832. 14965262

[ref8] Harvey A. L. Natural Products in Drug Discovery. Drug Discovery Today 2008, 13, 894–901. 10.1016/j.drudis.2008.07.004. 18691670

[ref9] Newman D. J.; Cragg G. M. Natural Products as Sources of New Drugs over the Nearly Four Decades from 01/1981 to 09/2019. J. Nat. Prod. 2020, 83, 770–803. 10.1021/acs.jnatprod.9b01285. 32162523

[ref10] Chugh R. M.; Mittal P.; Mp N.; Arora T.; Bhattacharya T.; Chopra H.; Cavalu S.; Gautam R. K. Fungal Mushrooms: A Natural Compound With Therapeutic Applications. Front. Pharmacol. 2022, 13, 925387 10.3389/fphar.2022.925387. 35910346 PMC9328747

[ref11] Tobert J. A. Lovastatin and beyond: The History of the HMG-CoA Reductase Inhibitors. Nat. Rev. Drug Discovery 2003, 2, 517–526. 10.1038/nrd1112. 12815379

[ref12] Strader C. R.; Pearce C. J.; Oberlies N. H. Fingolimod (FTY720): A Recently Approved Multiple Sclerosis Drug Based on a Fungal Secondary Metabolite. J. Nat. Prod. 2011, 74, 900–907. 10.1021/np2000528. 21456524

[ref13] Zhang R.; Yu S.; Bai H.; Ning K. TCM-Mesh: The Database and Analytical System for Network Pharmacology Analysis for TCM Preparations. Sci. Rep. 2017, 7, 2821 10.1038/s41598-017-03039-7. 28588237 PMC5460194

[ref14] Mohanraj K.; Karthikeyan B. S.; Vivek-Ananth R. P.; Chand R. P. B.; Aparna S. R.; Mangalapandi P.; Samal A. IMPPAT: A Curated Database of Indian Medicinal Plants, Phytochemistry And Therapeutics. Sci. Rep. 2018, 8, 4329 10.1038/s41598-018-22631-z. 29531263 PMC5847565

[ref15] van Santen J. A.; Jacob G.; Singh A. L.; Aniebok V.; Balunas M. J.; Bunsko D.; Neto F. C.; Castaño-Espriu L.; Chang C.; Clark T. N.; Cleary Little J. L.; Delgadillo D. A.; Dorrestein P. C.; Duncan K. R.; Egan J. M.; Galey M. M.; Haeckl F. P. J.; Hua A.; Hughes A. H.; Iskakova D.; Khadilkar A.; Lee J.-H.; Lee S.; LeGrow N.; Liu D. Y.; Macho J. M.; McCaughey C. S.; Medema M. H.; Neupane R. P.; O’Donnell T. J.; Paula J. S.; Sanchez L. M.; Shaikh A. F.; Soldatou S.; Terlouw B. R.; Tran T. A.; Valentine M.; van der Hooft J. J. J.; Vo D. A.; Wang M.; Wilson D.; Zink K. E.; Linington R. G. The Natural Products Atlas: An Open Access Knowledge Base for Microbial Natural Products Discovery. ACS Cent. Sci. 2019, 5, 1824–1833. 10.1021/acscentsci.9b00806. 31807684 PMC6891855

[ref16] Zeng X.; Zhang P.; Wang Y.; Qin C.; Chen S.; He W.; Tao L.; Tan Y.; Gao D.; Wang B.; Chen Z.; Chen W.; Jiang Y. Y.; Chen Y. Z. CMAUP: A Database of Collective Molecular Activities of Useful Plants. Nucleic Acids Res. 2019, 47, D1118–D1127. 10.1093/nar/gky965. 30357356 PMC6324012

[ref17] Vivek-Ananth R. P.; Mohanraj K.; Sahoo A. K.; Samal A. IMPPAT 2.0: An Enhanced and Expanded Phytochemical Atlas of Indian Medicinal Plants. 2022, bioRxiv:2022.06.17.496609 10.1021/acsomega.3c00156 PMC9996785 36910986

[ref18] Valverde M. E.; Hernández-Pérez T.; Paredes-López O. Edible Mushrooms: Improving Human Health and Promoting Quality Life. Int. J. Microbiol. 2015, 2015, 376387 10.1155/2015/376387. 25685150 PMC4320875

[ref19] Vivek-Ananth R. P.; Sahoo A. K.; Kumaravel K.; Mohanraj K.; Samal A. MeFSAT: A Curated Natural Product Database Specific to Secondary Metabolites of Medicinal Fungi. RSC Adv. 2021, 11, 2596–2607. 10.1039/d0ra10322e. 35424258 PMC8693784

[ref20] Medina-Franco J. L.; Martínez-Mayorga K.; Bender A.; Scior T. Scaffold Diversity Analysis of Compound Data Sets Using an Entropy-Based Measure. QSAR Comb. Sci. 2009, 28, 1551–1560. 10.1002/qsar.200960069.

[ref21] González-Medina M.; Prieto-Martínez F. D.; Owen J. R.; Medina-Franco J. L. Consensus Diversity Plots: A Global Diversity Analysis of Chemical Libraries. J. Cheminf. 2016, 8, 63 10.1186/s13321-016-0176-9. PMC5105260 27895718

[ref22] Khanna V.; Ranganathan S. Structural Diversity of Biologically Interesting Datasets: A Scaffold Analysis Approach. J. Cheminf. 2011, 3, 30 10.1186/1758-2946-3-30. PMC3179739 21824432

[ref23] Langdon S. R.; Brown N.; Blagg J. Scaffold Diversity of Exemplified Medicinal Chemistry Space. J. Chem. Inf. Model. 2011, 51, 2174–2185. 10.1021/ci2001428. 21877753 PMC3180201

[ref24] González-Medina M.; Owen J. R.; El-Elimat T.; Pearce C. J.; Oberlies N. H.; Figueroa M.; Medina-Franco J. L. Scaffold Diversity of Fungal Metabolites. Front. Pharmacol. 2017, 8, 180 10.3389/fphar.2017.00180. 28420994 PMC5376591

[ref25] González-Medina M.; Medina-Franco J. L. Chemical Diversity of Cyanobacterial Compounds: A Chemoinformatics Analysis. ACS Omega 2019, 4, 6229–6237. 10.1021/acsomega.9b00532.

[ref26] Naveja J. J.; Rico-Hidalgo M. P.; Medina-Franco J. L. Analysis of a Large Food Chemical Database: Chemical Space, Diversity, and Complexity. F1000Research 2018, 7, 993 10.12688/f1000research.15440.2. PMC6081979 30135721

[ref27] Al Sharie A. H.; El-Elimat T.; Al Zu’bi Y. O.; Medina-Franco A. J.; Medina-Franco J. L. Chemical Space and Diversity of Seaweed Metabolite Database (SWMD): A Cheminformatics Study. J. Mol. Graphics Modell. 2020, 100, 107702 10.1016/j.jmgm.2020.107702. 32810730

[ref28] El-Elimat T.; Zhang X.; Jarjoura D.; Moy F. J.; Orjala J.; Kinghorn A. D.; Pearce C. J.; Oberlies N. H. Chemical Diversity of Metabolites from Fungi, Cyanobacteria, and Plants Relative to FDA-Approved Anticancer Agents. ACS Med. Chem. Lett. 2012, 3, 645–649. 10.1021/ml300105s. 22993669 PMC3443637

[ref29] González-Medina M.; Prieto-Martínez F. D.; Naveja J. J.; Méndez-Lucio O.; El-Elimat T.; Pearce C. J.; Oberlies N. H.; Figueroa M.; Medina-Franco J. L. Chemoinformatic Expedition of the Chemical Space of Fungal Products. Future Med. Chem. 2016, 8, 1399–1412. 10.4155/fmc-2016-0079. 27485744 PMC5558535

[ref30] Wishart D. S.; Feunang Y. D.; Guo A. C.; Lo E. J.; Marcu A.; Grant J. R.; Sajed T.; Johnson D.; Li C.; Sayeeda Z.; Assempour N.; Iynkkaran I.; Liu Y.; Maciejewski A.; Gale N.; Wilson A.; Chin L.; Cummings R.; Le D.; Pon A.; Knox C.; Wilson M. DrugBank 5.0: A Major Update to the DrugBank Database for 2018. Nucleic Acids Res. 2018, 46, D1074–D1082. 10.1093/nar/gkx1037. 29126136 PMC5753335

[ref31] AnalytiCon Discovery. https://ac-discovery.com/screening-library-downloads/ (accessed 2022).

[ref32] Lipkus A. H.; Yuan Q.; Lucas K. A.; Funk S. A.; Bartelt W. F.; Schenck R. J.; Trippe A. J. Structural Diversity of Organic Chemistry. A Scaffold Analysis of the CAS Registry. J. Org. Chem. 2008, 73, 4443–4451. 10.1021/jo8001276. 18505297

[ref33] Lipkus A. H.; Watkins S. P.; Gengras K.; McBride M. J.; Wills T. J. Recent Changes in the Scaffold Diversity of Organic Chemistry As Seen in the CAS Registry. J. Org. Chem. 2019, 84, 13948–13956. 10.1021/acs.joc.9b02111. 31603683

[ref34] MeFSAT: Medicinal Fungi Secondary Metabolite And Therapeutics. https://cb.imsc.res.in/mefsat/ (accessed 2022).

[ref35] Sterling T.; Irwin J. J. ZINC 15 – Ligand Discovery for Everyone. J. Chem. Inf. Model. 2015, 55, 2324–2337. 10.1021/acs.jcim.5b00559. 26479676 PMC4658288

[ref36] Chambers J.; Davies M.; Gaulton A.; Hersey A.; Velankar S.; Petryszak R.; Hastings J.; Bellis L.; McGlinchey S.; Overington J. P. UniChem: A Unified Chemical Structure Cross-Referencing and Identifier Tracking System. J. Cheminf. 2013, 5, 3 10.1186/1758-2946-5-3. PMC3616875 23317286

[ref37] Ertl P.; Rohde B. The Molecule Cloud - Compact Visualization of Large Collections of Molecules. J. Cheminf. 2012, 4, 12 10.1186/1758-2946-4-12. PMC3403880 22769057

[ref38] Scopy. https://scopy.iamkotori.com/ (accessed 2022).

[ref39] Brown N.; Jacoby E. On Scaffolds and Hopping in Medicinal Chemistry. Mini-Rev. Med. Chem. 2006, 6, 1217–1229. 10.2174/138955706778742768. 17100633

[ref40] Leeson P. D.; Springthorpe B. The Influence of Drug-like Concepts on Decision-Making in Medicinal Chemistry. Nat. Rev. Drug Discovery 2007, 6, 881–890. 10.1038/nrd2445. 17971784

[ref41] Faller B.; Ottaviani G.; Ertl P.; Berellini G.; Collis A. Evolution of the Physicochemical Properties of Marketed Drugs: Can History Foretell the Future?. Drug Discovery Today 2011, 16, 976–984. 10.1016/j.drudis.2011.07.003. 21782967

[ref42] Sud M. MayaChemTools: An Open Source Package for Computational Drug Discovery. J. Chem. Inf. Model. 2016, 56, 2292–2297. 10.1021/acs.jcim.6b00505. 28024397

[ref43] Bemis G. W.; Murcko M. A. The Properties of Known Drugs. 1. Molecular Frameworks. J. Med. Chem. 1996, 39, 2887–2893. 10.1021/jm9602928. 8709122

[ref44] RDKit: Open-source cheminformatics. https://www.rdkit.org/ (accessed 2022).

[ref45] Godden J. W.; Bajorath J. Analysis of Chemical Information Content Using Shannon Entropy. Reviews in Computational Chemistry; John Wiley & Sons, Ltd, 2007; pp 263–289.

[ref46] Owen J. R.; Nabney I. T.; Medina-Franco J. L.; López-Vallejo F. Visualization of Molecular Fingerprints. J. Chem. Inf. Model. 2011, 51, 1552–1563. 10.1021/ci1004042. 21696145

[ref47] Lipinski C. A.; Lombardo F.; Dominy B. W.; Feeney P. J. Experimental and Computational Approaches to Estimate Solubility and Permeability in Drug Discovery and Development Settings. Adv. Drug Delivery Rev. 2001, 46, 3–26. 10.1016/s0169-409x(00)00129-0. 11259830

[ref48] Veber D. F.; Johnson S. R.; Cheng H.-Y.; Smith B. R.; Ward K. W.; Kopple K. D. Molecular Properties That Influence the Oral Bioavailability of Drug Candidates. J. Med. Chem. 2002, 45, 2615–2623. 10.1021/jm020017n. 12036371

[ref49] Egan W. J.; Merz K. M.; Baldwin J. J. Prediction of Drug Absorption Using Multivariate Statistics. J. Med. Chem. 2000, 43, 3867–3877. 10.1021/jm000292e. 11052792

[ref50] Perez J. J. Managing Molecular Diversity. Chem. Soc. Rev. 2005, 34, 143–152. 10.1039/b209064n. 15672178

[ref51] Karlov D. S.; Sosnin S.; Tetko I. V.; Fedorov M. V. Chemical Space Exploration Guided by Deep Neural Networks. RSC Adv. 2019, 9, 5151–5157. 10.1039/c8ra10182e. 35514634 PMC9060647

[ref52] Bishop C. M.; Svensén M.; Williams C. K. I. GTM: The Generative Topographic Mapping. Neural Comput. 1998, 10, 215–234. 10.1162/089976698300017953.

[ref53] Jolliffe I. T. Principal Component Analysis; Springer Series in Statistics; Springer New York: New York, NY, 1986.

[ref54] Bengio Y.; Courville A.; Vincent P. Representation Learning: A Review and New Perspectives. IEEE Trans. Pattern Anal. Mach. Intell. 2013, 35, 1798–1828. 10.1109/tpami.2013.50. 23787338

[ref55] Gaspar H. A. Ugtm: A Python Package for Data Modeling and Visualization Using Generative Topographic Mapping. J. Open Res. Software 2018, 6, 26 10.5334/jors.235.

[ref56] Pedregosa F.; Varoquaux G.; Gramfort A.; Michel V.; Thirion B.; Grisel O.; Blondel M.; Prettenhofer P.; Weiss R.; Dubourg V.; Vanderplas J.; Passos A.; Cournapeau D.; Brucher M.; Perrot M.; Duchesnay É. Scikit-Learn: Machine Learning in Python. J. Mach. Learn. Res. 2011, 12, 2825–2830.

[ref57] Hunter J. D. Matplotlib: A 2D Graphics Environment. Comput. Sci. Eng. 2007, 9, 90–95. 10.1109/mcse.2007.55.

