# Supplementary material for: Scaffold and Structural Diversity of the Secondary Metabolite Space of Medicinal Fungi
Source: ACS Omega. 2023 Jan 10;8(3):3102–13. doi: 10.1021/acsomega.2c06428 (PMC9878629; doi:10.1021/acsomega.2c06428)
Supplement: Supplementary file 1 — ao2c06428_si_001.pdf [file ao2c06428_si_001.pdf]

Supporting Information

Figures S1-S7

for

Scaffold and structural diversity of the secondary metabolite space of medicinal fungi

R.P. Vivek-Ananth[a,b], Ajaya Kumar Sahoo[a,b], Shanmuga Priya Baskaran[a,b], Areejit Samal[a,b,*]

[a] *The Institute of Mathematical Sciences (IMSc), Chennai 600113, India*

[b] *Homi Bhabha National Institute (HBNI), Mumbai 400094, India*

* To whom correspondence should be addressed: asamal@imsc.res.in

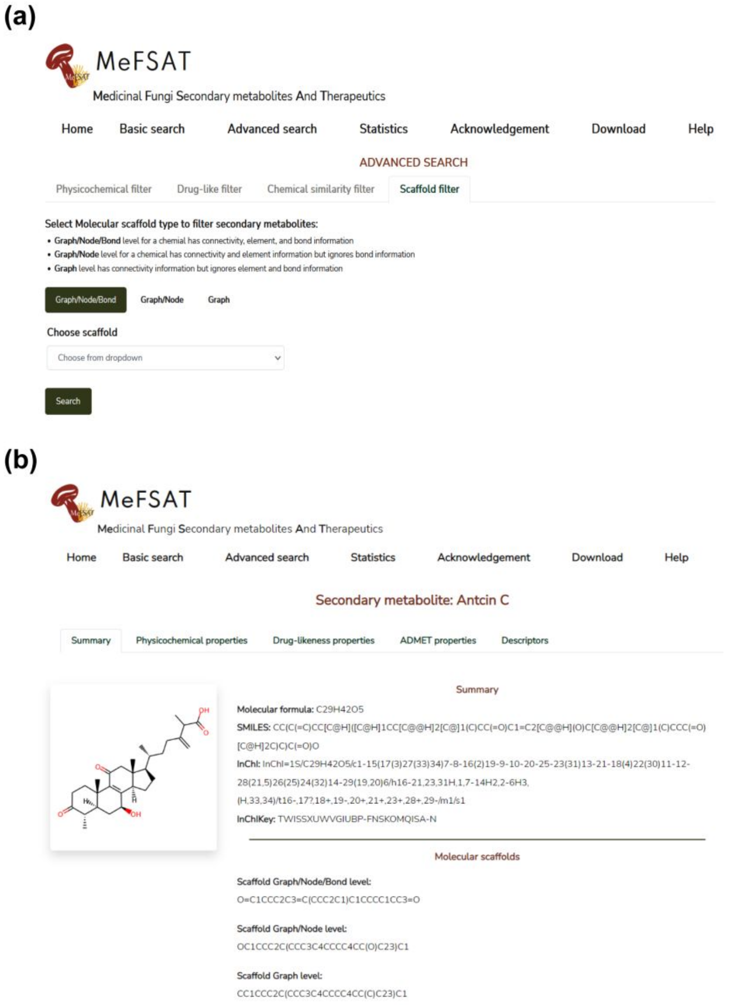

Figure S1: Screenshots of the **(a)** Scaffold filter tab under the Advanced Search option in the updated MeFSAT database to filter secondary metabolites by selecting scaffolds of interest, and **(b)** the detailed information page for a secondary metabolite in the updated MeFSAT database displaying the identified scaffolds for the secondary metabolite.

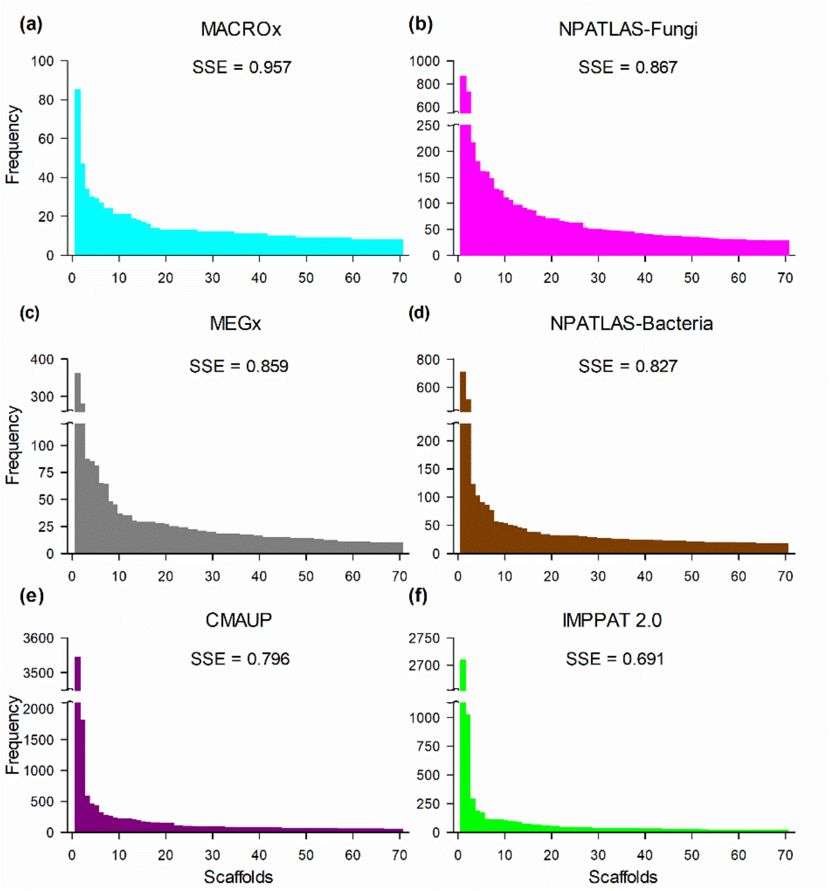

Figure S2: Distribution of chemicals across the top 70 most populated scaffolds in libraries: (a) MACROx, (b) NPATLAS-Fungi, (c) MEGx, (d) NPATLAS-Bacteria, (e) CMAUP, and (f) IMPPAT 2.0.

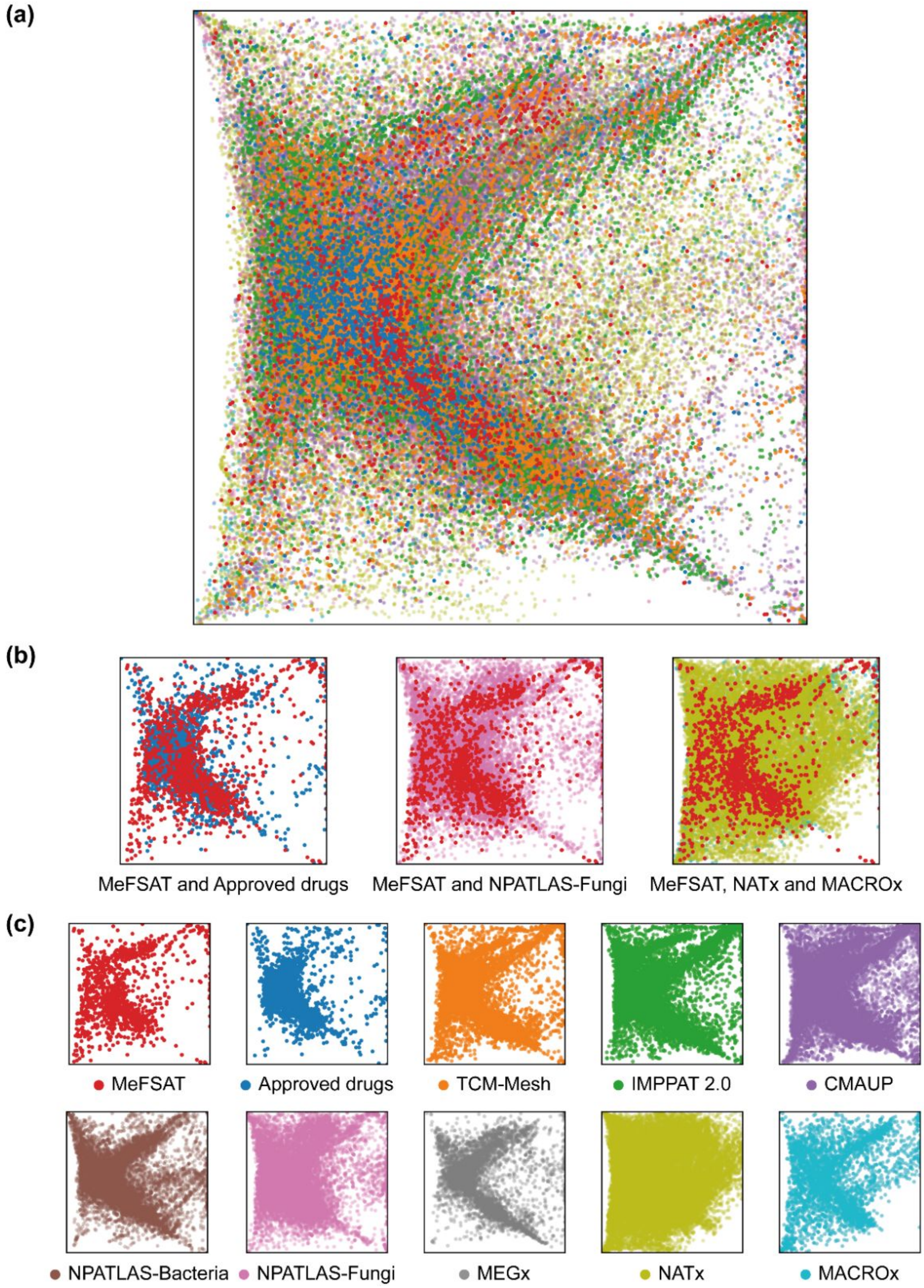

Figure S3: Visualization of the chemical spaces generated via GTM using molecular properties for the libraries analysed here. (a) Visualization of all chemical libraries analysed here. (b) Visualization of MeFSAT and Approved drugs, MeFSAT and NPATLAS-Fungi, and MeFSAT, NATx and MACROx. (c) Visualization of each individual chemical library. The colour used to represent each chemical library in the visualization is provided in part (c) along with the corresponding library name.

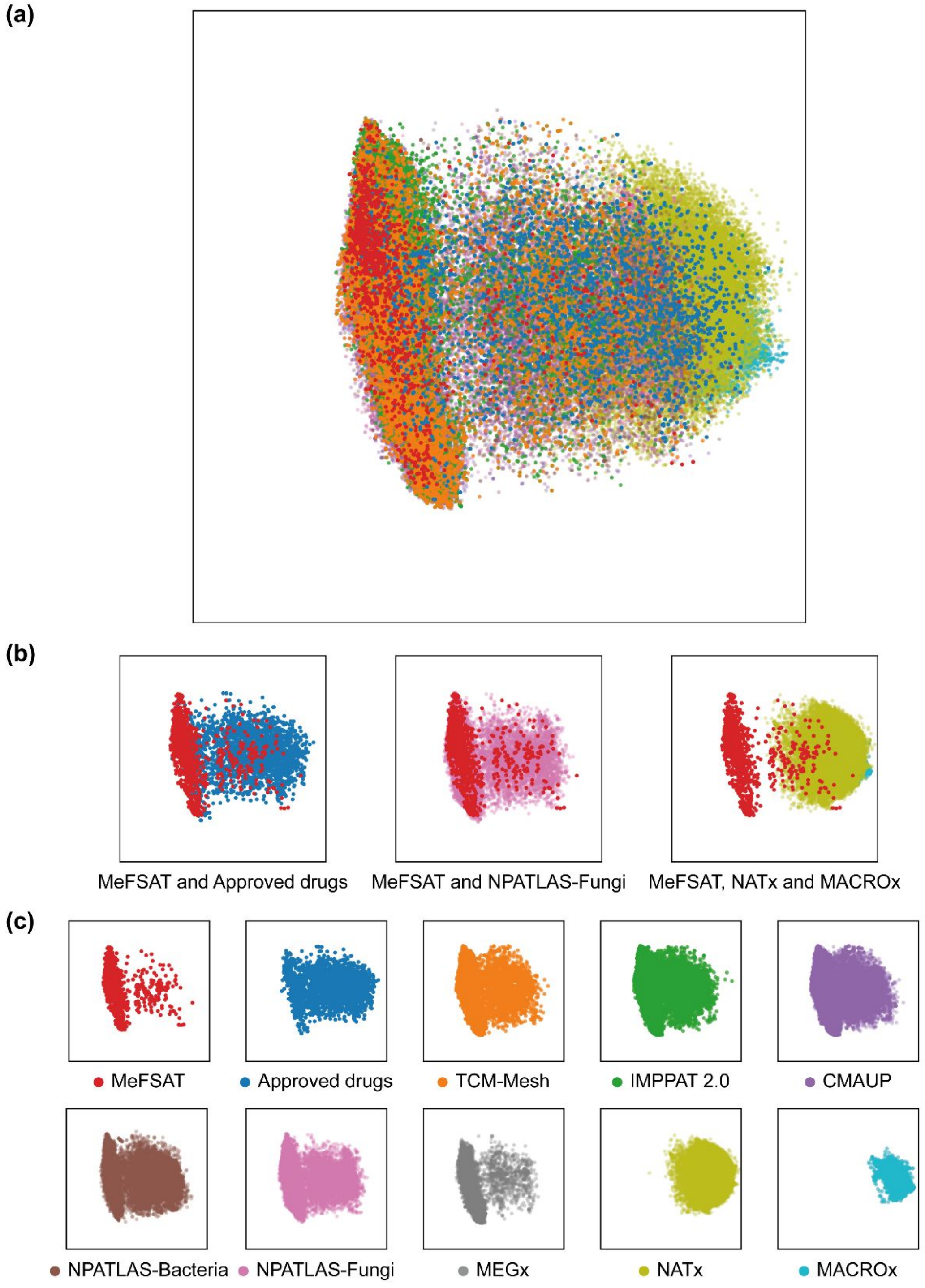

Figure S4: Visualization of the chemical spaces generated via PCA using MACCS keys structural fingerprints for the libraries analysed here. (a) Visualization of all chemical libraries analysed here. (b) Visualization of MeFSAT and Approved drugs, MeFSAT and NPATLAS-Fungi, and MeFSAT, NATx and MACROx. (c) Visualization of each individual chemical library. The colour used to represent each chemical library in the visualization is provided in part (c) along with the corresponding library name.

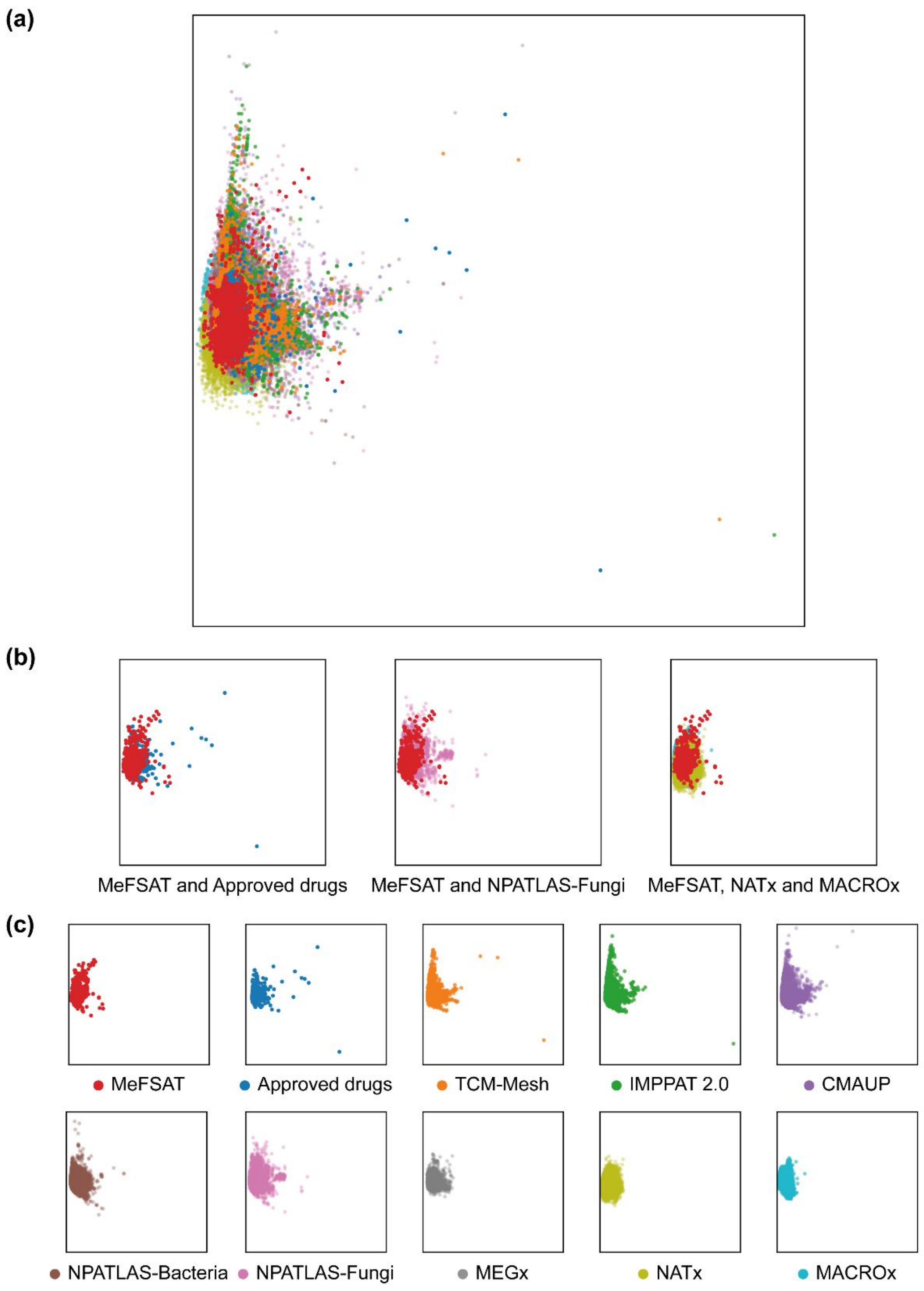

Figure S5: Visualization of the chemical spaces generated via PCA using molecular properties for the libraries analysed here. (a) Visualization of all chemical libraries analysed here. (b) Visualization of MeFSAT and Approved drugs, MeFSAT and NPATLAS-Fungi, and MeFSAT, NATx and MACROx. (c) Visualization of each individual chemical library. The colour used to represent each chemical library in the visualization is provided in part (c) along with the corresponding library name.

	MeFSAT	Approved drugs	TCM-Mesh	IMPPAT 2.0	CMAUP	NPATLAS-Bacteria	NPATLAS-Fungi	MEGx	NATx	MACROx
**MeFSAT**	*NA*									
**Approved drugs**	8	*NA*								
**TCM-Mesh**	82	181	*NA*							
**IMPPAT 2.0**	80	175	3423	*NA*						
**CMAUP**	130	248	5945	6703	*NA*					
**NPATLAS-Bacteria**	5	19	50	112	184	*NA*				
**NPATLAS-Fungi**	1202	10	102	121	451	2	*NA*			
**MEGx**	26	43	499	703	1058	59	199	*NA*		
**NATx**	0	0	0	0	0	0	0	0	*NA*	
**MACROx**	0	0	0	0	0	0	0	0	0	*NA*

Figure S6: Compound overlap between the chemical libraries analysed in this study. The triangular matrix provides the number of compounds common between different pairs of chemical libraries.

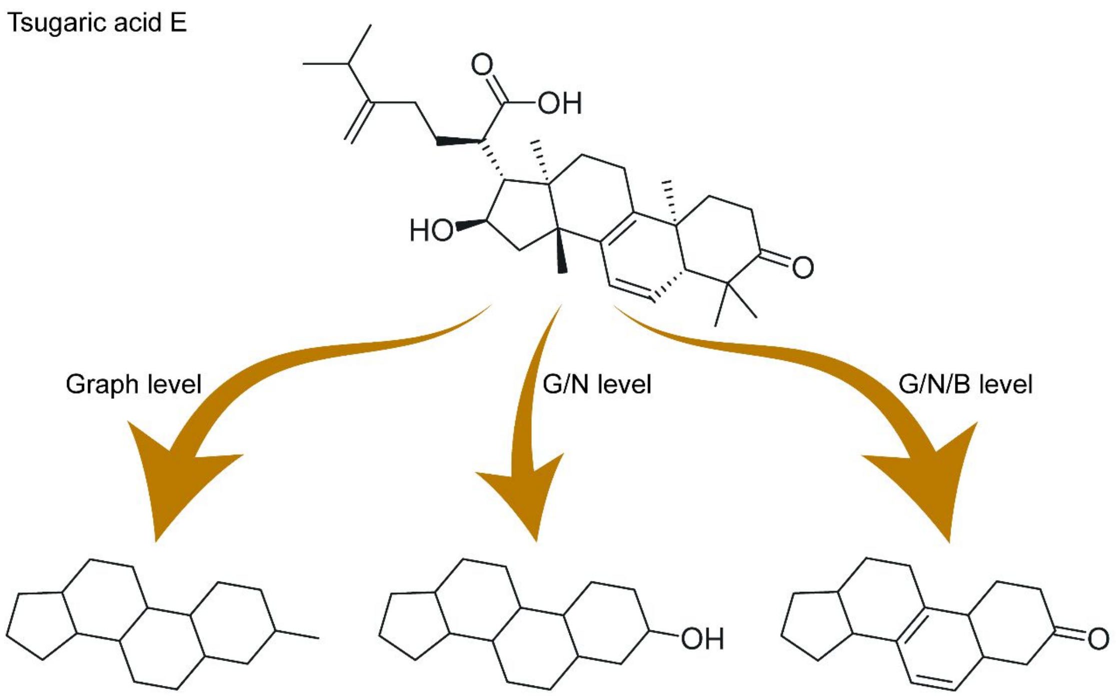

Figure S7: Molecular scaffold of the secondary metabolite Tsugaric acid E at three different levels: graph/node/bond (G/N/B) level (with connectivity, element and bond information), graph/node (G/N) level (with both connectivity and element information) and graph level (with only connectivity information).
